# Numerical Sensitivity Analysis for Dielectric Characterization of Biological Samples by Open-Ended Probe Technique

**DOI:** 10.3390/s20133756

**Published:** 2020-07-04

**Authors:** Marta Cavagnaro, Giuseppe Ruvio

**Affiliations:** 1 Department of Information Engineering, Electronics, and Telecommunications, Sapienza University, Piazzale Aldo Moro 5, 00185 Rome, Italy; 2 School of Medicine, National University of Ireland Galway, University Road, H91 TK33 Galway, Ireland; guiseppe.ruvio@nuigalway.ie; 3 Endowave Ltd., Dublin 2, Ireland

**Keywords:** dielectric properties measurement, open-ended probe, electromagnetic simulation

## Abstract

Dielectric characterization of biological tissues has become a fundamental aspect of the design of medical treatments based on electromagnetic energy delivery and their pre-treatment planning. Among several measuring techniques proposed in the literature, broadband and minimally-invasive open-ended probe measurements are best-suited for biological tissues. However, several challenges related to measurement accuracy arise when dealing with biological tissues in both ex vivo and in vivo scenarios such as very constrained set-ups in terms of limited sample size and probe positioning. By means of the Finite Integration Technique in the CST Studio Suite^®^ software, the numerical accuracy of the reconstruction of the complex permittivity of a high water-content tissue such as liver and a low water-content tissue such as fat is evaluated for different sample dimensions, different location of the probe, and considering the influence of the background environment. It is found that for high water-content tissues, the insertion depth of the probe into the sample is the most critical parameter on the accuracy of the reconstruction. Whereas when low water-content tissues are measured, the probe could be simply placed in contact with the surface of the sample but a deeper and wider sample is required to mitigate biasing effects from the background environment. The numerical analysis proves to be a valid tool to assess the suitability of a measurement set-up for a target accuracy threshold.

## 1. Introduction

In recent years, the rapid growth of medical applications based on electromagnetic (EM) fields such as hyperthermic treatments [1,2,3], medical imaging [4], on-body and implant-based communications [5] has focused a strong interest in the accurate dielectric characterization of biological tissues. Dielectric properties of tissues mediate the interaction between EM fields and the human body, and their values as well as the way these values change e.g., as a function of frequency, tissue status, temperature, are critical in the different applications [3,6]. This is particularly evident when numerical models are used e.g., to design new applications or to develop treatment planning procedures [7,8,9]. Accordingly, enhanced knowledge of the dielectric properties of human tissues and how these parameters change with time, temperature, hydration, and blood perfusion can inform the design of safer and more efficacious therapeutic, diagnostic and theranostic EM-based devices.

It was in 1984 when Joines [10] reported that diseased tissues show different dielectric values from healthy ones, giving momentum to the search of new techniques based on interaction of EM fields with the biological tissues. Dielectric spectroscopy has been facilitated by a deeper understanding of the interaction of complex biological tissues and EM energy across different frequency, power and duration settings. Extensive literature reviews of dielectric properties of biological tissues have been carried out over a wide frequency range [11]. Earlier studies mainly reported dielectric properties of animal tissues [12,13,14]; starting from 2000’s the focus of several studies has shifted towards human tissues [15]. Tissue properties datasets have been built from both in vivo and ex vivo measurements for animal tissues and ex vivo measurements for human tissues [16,17,18,19,20]. At microwave frequencies, the dominant and divergent components of the dielectric dispersion in biological tissues are the dipolar relaxation of water and the insulating behaviour of fat [13]. This defines the range of variability of dielectric properties in biological tissue within the limit conditions of fat tissue and highly-perfused tissues with high-water content. For this reason, the dielectric characterisation can be used to monitor thermal ablation treatments which induce loco-regional coagulative necrosis and a consequent dramatic dehydration of the targeted body region [6,21].

Recent studies have also started to address the impact of heterogeneity of biological tissues on dielectric properties measurements [22,23]. To this extent, it was proposed to cross-correlate the histological characterization of the volume of tissues that contribute to the dielectric properties measured by the probe. However, while improved protocols for the characterization of heterogeneous biological tissue are investigated, research efforts are also required to make the measurement set-up minimally invasive.

Non-destructive techniques are required to measure the dielectric properties of biological tissue samples to mitigate the risk of alteration of the tissue structure. There are several methods to measure the dielectric properties of biological tissues, including the transmission line, cavity, tetrapolar (or multi electrode) probe, and open-ended coaxial probe techniques [24,25,26]. Amongst these methods, the coaxial probe technique is the most commonly used [27,28]. The technique was extensively investigated and used not only to measure biological tissues but also dielectric for microwave circuits [29,30,31,32].

The open-ended coaxial probe consists of a truncated section of a transmission line. The electromagnetic field propagates along the coaxial line and reflection occurs when the electromagnetic field encounters an impedance mismatch between the open tip of the probe and the tissue sample. The reflected signals at different frequencies are measured and then converted into complex permittivity values. This broadband and minimally-invasive measurement technique is best-suited to measure semi-solid, pliable-solid, and liquid tissue samples [33,34] and can be potentially minimally invasive if the cross-section of the probe interfacing with the tissue is kept small in terms of the sample size. Stuchly and Stuchly extensively studied the open-ended probe method both theoretically and experimentally [35]. They made a critical review of coaxial-line reflection methods and started building a database with the dielectric properties of numerous tissues in the frequency range 10 kHz–10 GHz.

The accuracy of the open-ended probe measurement technique strongly depends on the probe’s dimensions and the frequency employed, as well as the position of the probe and the size of the sample in term of the wavelength in the unknown medium. In particular, when the boundaries of the samples are too close to the probe, the measurement can be strongly biased. For instance, according to the Keysight N1501A Dielectric Slim-form Probe Kit manual, the slim form probe should be inserted into the material under test at least for 5 mm, and 5 mm minimum are needed around the tip of the probe [34]. However, these operating conditions may be very challenging when dealing with biological tissues in both ex vivo and in vivo scenarios where the size of the specimen is often smaller than minimum requirements and the probe may not be inserted at recommended depth.

In this numerical investigation the impact on accuracy of the sample size and the probe positioning will be calculated for different conditions and for two reference tissues representing very different water-content levels (i.e., liver and fat). In particular, liver and fat tissues were used as reference for two main reasons: they are representative of the range of variability of dielectric properties in biological tissue, being the first a high water content tissue (about 70% [36]) and the latter a low-content one (about 18% [36]), i.e., two very distinct cases encountered when measuring dielectric properties of biological tissues; and these two tissues represent very often the target scenario in some of the above cited clinical applications (e.g., hyperthermia and microwave thermal ablation).

Aim of this paper is to introduce an accurate numerical model to validate the measurement set-up in terms of the expected complex permittivity of the medium under test. Moreover, once the numerical model is benchmarked against reference data from literature, it enables the analysis of the influence of the dimension of the sample under test on the accuracy of the reconstruction. Section 2 describes the numerical model and solver adopted for this investigation. But it also illustrates the methodology proposed for the sensitivity analysis of the reconstruction accuracy for different set-up configurations. Results of the numerical analysis carried out on liver and fat tissue are reported in Section 3 and extensively discussed in Section 4. Finally, in the last section, conclusions are drawn on the validity and the applicability of the proposed approach.

## 2. Materials and Methods

In this section the numerical method used in the analysis is described first. Then, to allow understanding the geometry implemented and simulated into the software, the experimental set-up for the measurement of dielectric properties of biological tissue by means of the open-ended probe technique is briefly recalled. The conducted sensitivity analysis is finally presented at the end of the section.

### 2.1. Numerical Method and Model of the Open-Ended Probe Technique Experimental Set-Up

CST Studio Suite^®^ software (Dassault Systèmes, Vélizy-Villacoublay, France) was used to perform numerical simulations. CST Studio Suite^®^ is a full-wave electromagnetic software suitable for the design, analysis and optimization of electromagnetic components. In the software, several numerical methods can be chosen to solve Maxwell’s equation, according to the problem at hand. In this work, the Transient Solver was used, that allows the calculation of the electromagnetic field in a broad frequency range. The Transient Solver implements the Finite Integration Technique to solve Maxwell’s equations in the time domain [37]. To this end, a mesh is used to discretize the domain under study. Both hexahedral and tetrahedral meshes can be used; moreover, several techniques are implemented to deal with boundaries which do not conform to the cells’ geometry [38].

Into the software a model of the experimental set-up used in the measurement of dielectric properties of biological tissues by way of the open-ended coaxial probe technique was implemented.

The technique is based on the measurement by a vector network analyser (VNA) of the reflection coefficient of a section of coaxial cable open-terminated (open-ended probe), when the tip is immersed in the material under test (MUT). The unknown dielectric properties are then calculated starting from the reflection coefficients and taking advantage of an equivalent circuit representing the probe as well as the MUT from an electric point of view [35,39,40].

Before the measurement in the MUT, a calibration procedure is performed to rule out systematic errors, as well as to characterize the elements of the equivalent circuit [12,39,41]. Stuchly et al. proposed a calibration technique using three standard loads - an open circuit, a short circuit and a liquid with known dielectric properties - which generate known reflection coefficients at the end of the line [12,42]. Recently, a calibration procedure made by using an open circuit condition and two well-characterized liquids has been proposed also, to allow reducing the uncertainty linked to the realization of the short condition at the terminal end of the coaxial cable [40,41].

After calibrating the set-up, the experimental procedure foresees the insertion of the open-ended probe into the MUT and the measurement of the resulting frequency dependent complex reflection coefficient (magnitude and phase). Then, from the measured data, inversion techniques are used to derive the unknown dielectric properties [40].

Several open-probes have been developed, both custom-made [41,43] and commercial ones [33,34]. In this study, the Keysight slim-form probe [33,34] was taken as a reference in terms of dimensions and applicability requirements, being one of the most frequently used probes in the experimental studies [44,45]. The Keysight slim-form probe is made by a 20 cm long section of coaxial cable, with an external diameter of 2.2 mm. According to the Keysight’s manual, the slim form probe should be used to measure liquid or semi-solid materials which could conform to the probe tip [34]. It operates in a frequency range between 500 MHz and 50 GHz (the maximum frequency is limited by MUT properties), and it should be inserted into the MUT at least for 5 mm, with 5 mm around the tip of the probe [34]. These conditions should approximate a material “infinite” in size, which is one of the hypotheses of the reconstruction models. Moreover, the MUT is supposed to be homogeneous and isotropic [33].

In the CST software, a section of coaxial cable 10 cm long open terminated in one end and excited with the fundamental TEM mode on the other end was modelled. Cable dimensions and materials were defined in order to conform to the slim-form structure (i.e., inner conductor 0.7 mm diameter, outer conductor inner diameter 1.8 mm and external diameter 2.2 mm, and relative permittivity of dielectric 1.28 to achieve 50 Ω impedance). Finally, no flange is present.

Simulations were conducted defining an initial mesh with a side of λ/30, where λ is the lowest wavelength in the simulation, and defining local mesh properties at the open tip of the probe imposing an edge refining factor of 10. Then simulations were carried out activating the adaptive mesh refinement process, with convergence criteria defined by the S-parameter values in the full frequency range of the simulation (0–5 GHz). Following the adaptive process, a mesh requirement of λ/40 in the domain, with the refinement factor of 10 at the edge of the probe was found as the optimal choice for accurate and stable results. The boundary conditions settled in the simulations where the “open boundary conditions” asking for at least 1 additional wavelength of space (at 1.5 GHz) around the studied geometry, with an estimated reflection level at the boundary of 0.0001.

To reconstruct the dielectric properties of materials, the model proposed by Stuchly&Stuchly (in the following reported as S&S’s model) representing the probe’s load as an admittance made by capacitive components, was used [12,35]. The model shows a good accuracy in the frequency range between 100 MHz and 5 GHz [35,46]. The high frequency limit is given by the increasing influence of radiation, and the low frequency limit by a decreasing sensitivity of the load capacitance model for varying permittivity [31,35].

Before considering any biological tissue, three distinct simulations were carried out leaving the tip of the probe in air, or inserting it into distilled water and methanol. Then the simulation was carried out with the probe inserted in the material under test, and the calculated reflection coefficient used to reconstruct the unknown dielectric properties.

The materials under test were liver and fat, representatives of a high and low water content tissue [36], i.e., two very distinct cases encountered when measuring dielectric properties of biological tissues. Their properties were implemented into the software according to the Debye’s dispersion model, i.e., taking into account the frequency-dependence of the dielectric properties of the two tissues [17].

The simulated frequency band was between 0 and 5 GHz, which covers most of medical applications of electromagnetic fields as microwave imaging, hyperthermia, thermal ablation [1,2,3].

To evaluate the error introduced by reducing the sample’s dimensions or the insertion depth of the probe, the general rules reported in the Keysight manual were taken as a reference [34]. In particular, according the manual, the model accuracy is typically about 3% to 5% for both the real and imaginary part of the relative complex permittivity; then, the overall measurement accuracy is calculated adding this data to the accuracy in reconstructing the calibration loads, reaching typical values of 10% in the case of the slim form probe [34].

Accordingly, in the following analysis of the achieved results, a threshold of 5% has been chosen to comment on the accuracy of the reconstruction of the unknown dielectric properties.

### 2.2. Sensitivity Analysis

**Figure 1:**
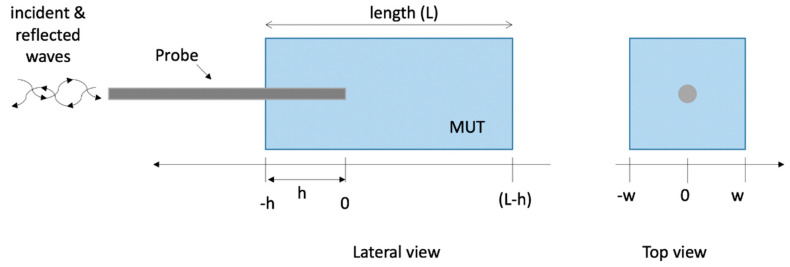
Geometry of the probe inserted into a parallelepiped of material (distilled water for calibration purposes, or liver and fat tissues).

As initial condition, both calibration liquids (i.e., distilled water and methanol) and the MUT were represented as a 7 cm long parallelepiped, with a square base of 3 cm side. The probe was inserted into the materials in the centre of their base, for a 2 cm length. Figure 1 shows the corresponding geometry.

**Figure 2:**
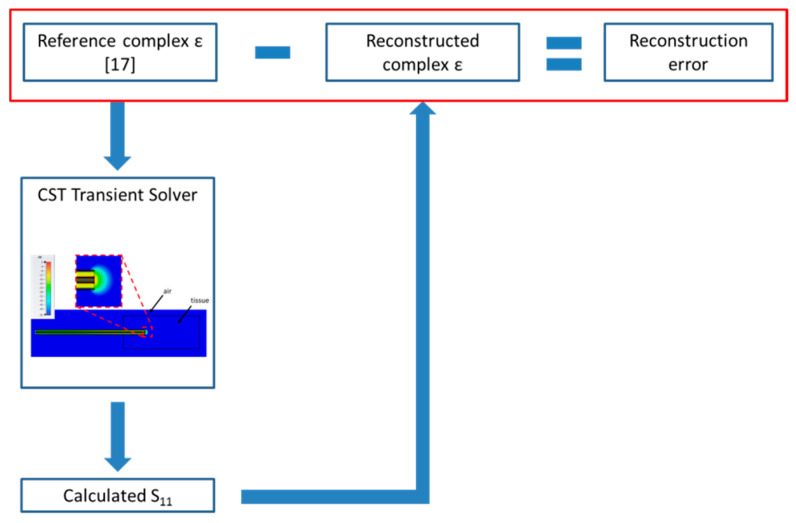
Flow diagram showing reconstruction error calculation.

The dielectric properties of the MUT as reconstructed with the S&S’s model were then compared with the values implemented in the CST software to validate the accuracy both of the simulations and of the reconstruction model. In particular, the comparison of the calculated data with the true values in the reference condition allows to validate the numerical model in terms of MUT dimensions, studied domain dimensions, and software settings. Figure 2 shows a schematic view of the adopted procedure.

Then, the sensitivity analysis was conducted reducing the dimensions of the MUT (length L and width w, Figure 1) as well as the insertion depth of the probe (h, Figure 1). The presence of a support to hold the sample under test was considered also simulating the lifting tables used in experimental conditions to press the biological sample against the probe tip in order to make a good contact without moving the probe after calibration [47]; in the simulations, the support was modelled as a perfect electric conductor (PEC) placed below the MUT.

Table 1 shows all cases considered in the sensitivity analysis. Starting from the reference condition (height 70 mm, width 15 mm—i.e., 30 mm sample’s side, probe insertion depth 20 mm, background material air), the height of the sample was reduced first, then its width. Both these two cases (2 and 3, Table 1) maintain the measurement conditions reported in the Keysight’s manual, i.e., 5 mm insertion depth and 5 mm tissue all around the probe tip [34]. Then, the insertion depth of the probe was nullified both with the reference dimensions and with reduced height and width of the sample (4 and 5, Table 1).

Successively, the sample height was gradually reduced keeping the minimum allowable sample’s width and probe insertion depth (6–10, Table 1). From the data in the Table, for case 10, a distance of 1 mm between the probe’s tip and the bottom surface of the sample can be calculated (L–h). Similarly, keeping the smallest allowable height and insertion depth of the probe, the sample width was gradually reduced (11–13, Table 1). Then, the insertion depth was varied, reducing the height of the sample and the insertion depth at the same time, in order to keep the distance between the tip of the probe and the bottom surface of the sample constant (i.e., 5 mm; cases 14–18, Table 1).

Finally, the presence of the lifting table used to hold the sample and to lift it was considered, backing the bottom surface of the sample with a perfect electric conductor. The presence of the PEC was considered in the case of sample and measurement conditions equal to the minimum requirements (19, Table 1) and with reduced distance between the probe’s tip and the sample’s bottom surface, still keeping the 5 mm insertion depth (20, Table 1).

The accuracy of the reconstruction in the different cases was evaluated calculating the percentage error at each considered frequency according to Equation (1):

(1) $$\frac{\left|X_{CST}\left(f\right)-X_{sim}\left(f\right)\right|}{X_{CST}\left(f\right)}\cdot 100,$$

where $X_{CST}$ represents either the real part ($\varepsilon^{\prime }$) or the imaginary part ($\varepsilon^{\prime \prime }$) of the complex relative permittivity of the MUT implemented in the CST software, i.e., the actual values for this study, $X_{sim}$ the corresponding quantity calculated from the simulations, and f represents the frequency. In total 980 frequency points were considered in the range from 100 MHz to 5 GHz, corresponding to a frequency resolution of 5 MHz.

Among all frequency values, average values and standard deviations where then calculated.

## 3. Results

In the following, the results achieved in the reference condition are presented first. Then, the results achieved changing the dimensions of the MUT are presented, both with reference to liver and to fat tissue.

### 3.1. Reference Condition

**Figure 3:**
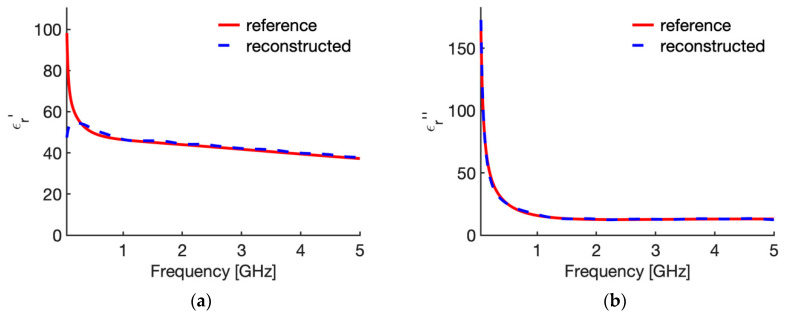
Relative complex permittivity of liver at 25 °C. (**a**) real part; (**b**) imaginary part. The values implemented into the CST software (red line) are compared with those reconstructed by the Stuchly’s model (dashed blue line) from the simulated reflection coefficient of the open probe.

**Figure 4:**
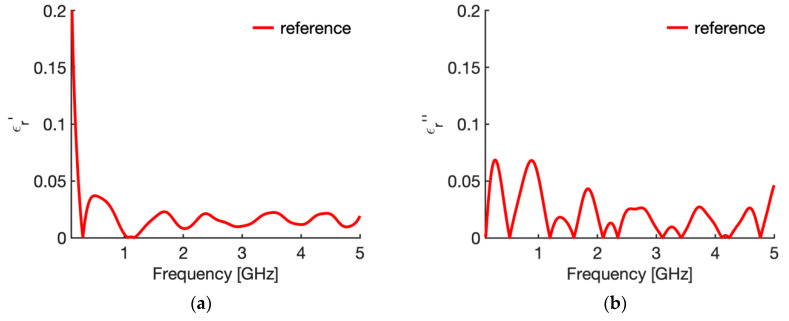
Percentage errors in the evaluation of the relative complex permittivity of liver at 25 °C through the reflection arrangement of the open probe technique (**a**) real part; (**b**) imaginary part.

**Figure 5:**
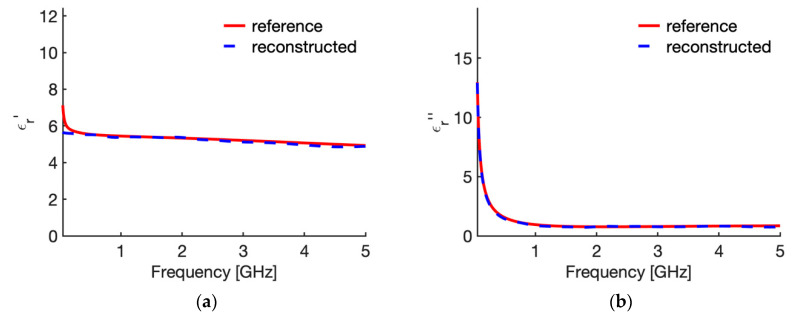
Relative complex permittivity of fat at 25 °C. (**a**) real part; (**b**) imaginary part. The values implemented into the CST software (red line) are compared with those reconstructed by the Stuchly’s model (dashed blue line) from the simulated reflection coefficient of the open probe.

**Figure 6:**
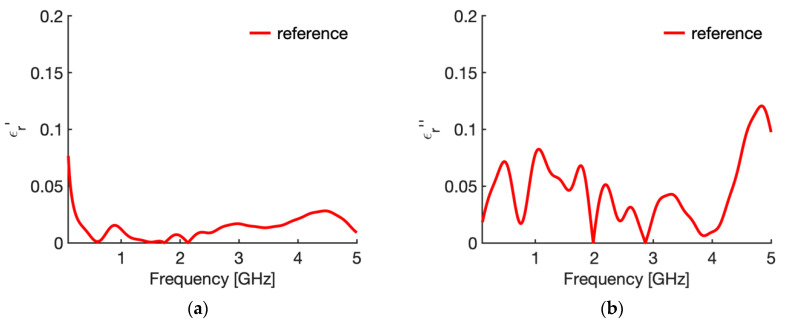
Percentage errors in the evaluation of the relative complex permittivity of fat at 25 °C through the reflection arrangement of the open probe technique. (**a**) real part; (**b**) imaginary part.

Figure 3 shows the real (Figure 3a) and imaginary part (Figure 3b) of the relative complex permittivity of liver at 25 °C as a function of the frequency as reconstructed by the Stuchly model compared with the values in the CST software (i.e., the actual ones for this study). To evidence the accuracy of the reconstruction, Figure 4 shows the corresponding percentage error. Similarly, Figure 5 compares the real (Figure 5a) and imaginary part (Figure 5b) of the relative complex permittivity of fat at 25 °C as a function of the frequency reconstructed by the Stuchly model with the values in the CST software, and Figure 6 shows the corresponding percentage errors.

From the figures an optimum agreement between the actual values and those reconstructed can be derived in the frequency range of the probe, i.e., from 0.5 GHz up to 5 GHz. In particular, Figure 3 and Figure 5 evidence the frequency applicability of the reconstruction model, showing increasing differences in the reconstructed values for frequency values below 100 MHz. Indeed, as stated in Section 2.1, at the lower frequencies the model loses sensitivity due to too small changes of the admittance load linked to different MUT. Looking at the percentage errors, i.e., Figure 4; Figure 6, in the considered frequency range an oscillating behaviour with the frequency is found, with maximum values below 0.04 and 0.07 for the real and imaginary part of the complex permittivity of liver (Figure 4), and below 0.03 and 0.1 for the real and imaginary part of the complex permittivity of fat (Figure 6), with the exception of the imaginary part at the highest frequencies (Figure 6b). In this respect, it can be noted that the slim form probe is not recommended for measurement of materials with low loss tangent (loss tangent < 0.5) when the real part of the complex permittivity is greater than 5 [34]. In the case of fat, the loss tangent is below 0.5 from about 250 MHz, and the real part of the complex permittivity is greater than 5 up to 4.5 GHz, and equal to 4.93 at 5 GHz. Accordingly, the considered material shows dielectric properties which are at the limit of the usability of the open-ended probe technique with the probe’s dimensions here considered. However, the literature shows that the advantages of the open-ended probe technique in terms of minimal invasiveness and broad frequency range have induced its large adoption also at limit conditions for the dielectric characterization of biological tissues with low water content [10,11,12,13,14,15,16,17,18,19,27,28,35,43,44,45].

From Figure 3, Figure 4, Figure 5 and Figure 6 it can be noted that a good agreement is kept even for frequencies lower than 0.5 GHz; following this result, the percentage errors and related statistics were evaluated starting from 100 MHz. In the case of liver, the average percentage error is 1.9% (st. dev. 2.0) for the real part and 2.1% (st. dev. 1.7) for the imaginary part of the complex permittivity; while in the fat the percentage error is 1.3% (st. dev. 1.0) and 4.6% (st. dev. 2.9), respectively. As a matter of fact, differences below 5% of the actual values are found from 360 MHz in the case of liver and from 140 MHz in the case of fat.

### 3.2. Sensitivity Analysis

Table 2 reports the average values and standard deviations of the error in the frequency range from 100 MHz to 5 GHz for all considered cases when the MUT is liver, while Table 3 shows the same data in the case of fat. The column “Case” in the Tables reports a short reference to the considered case, as detailed in Table 1, given by an explicative name followed in parentheses by the geometrical dimensions (height, width, insertion depth of the probe) and backing material (i.e., air or Perfect Electric Conductor, PEC).

In the Tables, values in red colour evidence the errors which are above a value of 5%.

From Table 2; Table 3, it can be noted that the average errors evaluated for the imaginary part of the complex permittivity are always greater than the related errors for the real part.

Comparing the reference cases with all others, it can be noted that the minimum dimensions and probe insertion usually considered in experimental studies (5 mm insertion depth, and 5 mm distance between the probe and the surfaces of the MUT in all directions) increase the achievable average error from a minimum value of 1.9 to 2.8 for $\varepsilon^{\prime }$ and from 2.1 to 4.8 for $\varepsilon^{\prime \prime }$ in the case of liver, and from 1.3 to 1.6, from 4.6 to 8.1, respectively, in the case of fat (comparison of case 1 with case 6).

In the case of liver, shown in Table 2, calculated errors are below 5% in most considered cases with the exception of the cases in which the probe is just put in contact with the surface of the MUT (0 insertion, cases n. 4, 5, 18), when the height of the sample is reduced such that the distance between the probe tip and the bottom surface of the MUT is 2 mm or lower (cases 9 and 10), when the MUT width is 3 mm or lower (cases 12 and 13), and when the insertion of the probe is minimum (1 mm, case 17). Comparing cases 4 and 17, i.e., a big MUT but with the probe placed in contact to the upper surface (4) and a small MUT (17) with the probe inserted for 1 mm, so that the distance between the probe tip and the bottom surface of the sample is 5 mm, it can be noted that the errors in the real part of the complex permittivity are almost the same, while the error in the imaginary part is greater for the case with the big sample. Accordingly, it can be derived that the main source of error in evaluating the MUT complex permittivity is when the probe is not inserted into the MUT but simply put in contact with its surface. Finally, the greatest values for the average error are achieved in the case of reduced sample height, when the distance between the probe tip and the bottom surface of the MUT is greatly reduced (case 10), and when the width of the sample is reduced with respect to the probe dimensions (case 13). Considering a maximum allowable accuracy equal to 5%, it can be concluded that the sample height can be slightly reduced from 10 mm to 8 mm if the insertion depth of the probe is kept at least 5 mm, while the sample width should be at least 4 mm, i.e., very close the experimental indications [34]. However, if the insertion depth is reduced, even keeping the distance between the probe tip and the bottom surface of the MUT at 5 mm, the error increases above the threshold.

Finally, in both liver and fat, if the MUT is placed on a conductive support (cases 19 and 20), the error is slightly reduced with respect the case of the same geometrical conditions and background medium air, in both considered materials (6 vs. 19, and 15 vs. 20).

**Figure 7:**
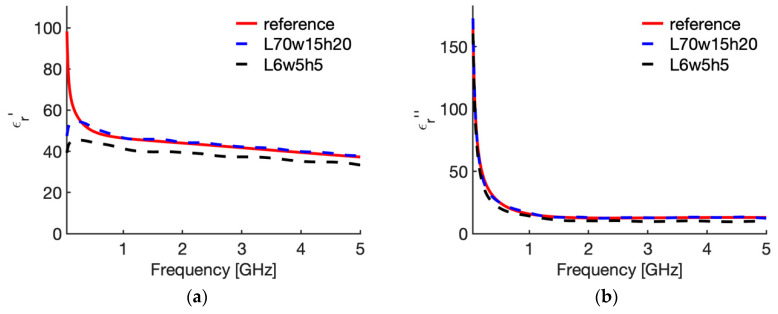
Relative complex permittivity of liver at 25 °C. (**a**) real part; (**b**) imaginary part. The values implemented into the CST software (red line) are compared with those reconstructed by the S&S model from the simulated reflection coefficient of the open probe in the reference case (dashed blue line), and in the case in which the maximum percentage error was evaluated (dashed black line, 6; 5; 5; air).

**Figure 8:**
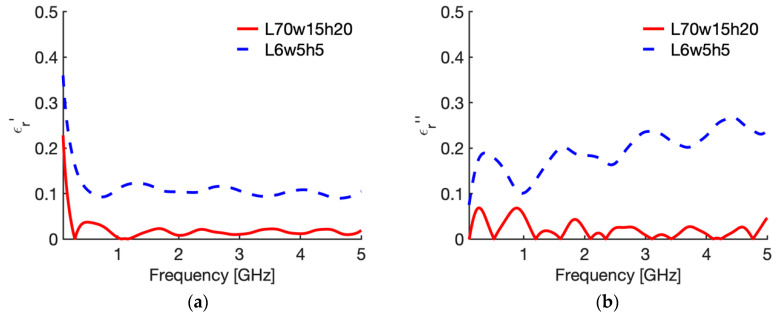
Percentage errors in the evaluation of the relative complex permittivity of liver at 25 °C through the reflection arrangement of the open probe technique. (**a**) real part; (**b**) imaginary part. The red curves give the error evaluated in the reference case, while the dashed blue line shows the error in the case where the maximum average error was calculated, i.e., case 10 in Table 2 (6; 5; 5; air).

Figure 7 shows the behaviour of the complex permittivity of liver (real part Figure 7a; imaginary part Figure 7b) comparing the “real values”, i.e., the data implemented in the CST software, with those reconstructed from the reflection coefficient of the probe in the reference case (70,15,20,a) and in the case where the maximum average error was calculated, i.e., case 10 in Table 2 (6,5,5,a). To evidence the distance between the “real values” and the reconstructed ones, Figure 8 shows the corresponding percentage error.

**Figure 9:**
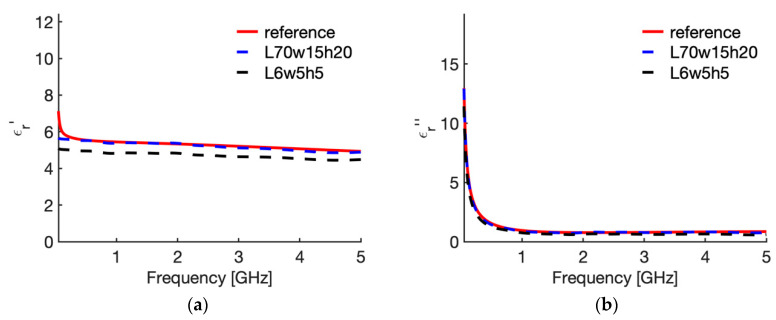
Relative complex permittivity of fat at 25 °C. (**a**) real part; (**b**) imaginary part. The values implemented into the CST software (red line) are compared with those reconstructed by the S&S model from the simulated reflection coefficient of the open probe in the reference case (dashed blue line), and in the case in which the maximum percentage error was evaluated (dashed black line, 6; 5; 5; air).

**Figure 10:**
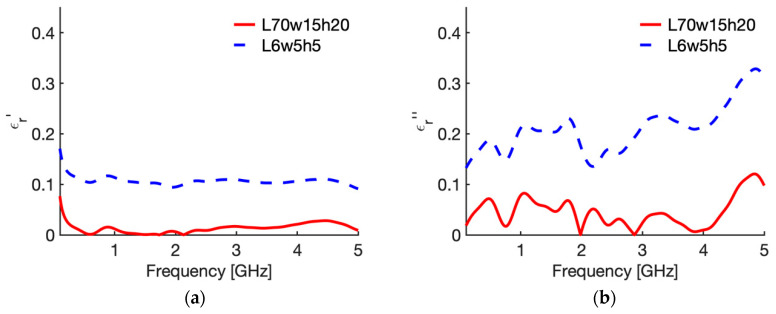
Percentage errors in the evaluation of the relative complex permittivity of fat at 25 °C through the reflection arrangement of the open probe technique. (**a**) real part; (**b**) imaginary part. The red curves give the error evaluated in the reference case, while the dashed blue line shows the error in the case where the maximum average error was calculated, i.e., case 10 in Table 2 (6; 5; 5; air).

Similarly, Figure 9 shows the behaviour of the complex permittivity of fat (real part Figure 9a; imaginary part Figure 9b) comparing the “real values” with those reconstructed from the reflection coefficient of the probe in the reference case (70,15,20,a) and in the case where the maximum average error was calculated, i.e., case 10 in Table 3 (6,5,5,a). Again, to evidence the distance between the “real values” and the reconstructed ones, Figure 10 shows the corresponding percentage error. From Figure 7 and Figure 9 it is shown that the error is due to lower values calculated with the reconstruction algorithm in all the frequency band considered, with a slight increase in the upper frequencies for the imaginary part. Accordingly, the calculated error behaves as a systematic error with, in the worst case (i.e., 6; 5; 5; air), an average weight across all frequencies equal to 11.1% for ε′ and 19.6% for ε″ in the case of liver, and 10.7% and 21.0% for ε′ and ε″ in the case of fat (Table 2 and Table 3, case 10).

**Figure 11:**
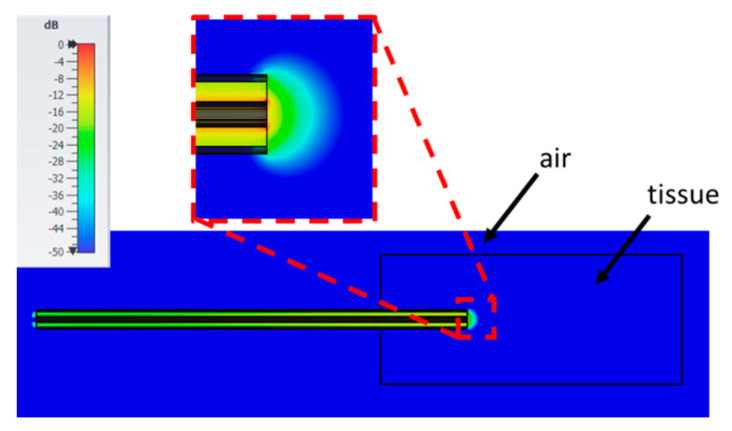
RMS electric field at 0.5 GHz evaluated in the fat in the reference case (70, 15, 20, a). Colour scale in dB_max_ (0; −50 dB).

**Figure 12:**
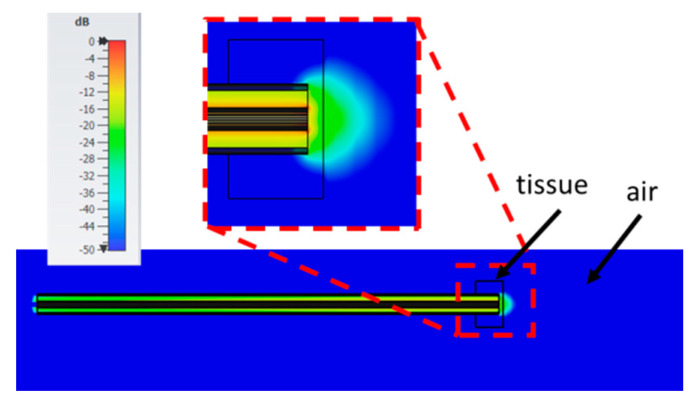
RMS electric field at 0.5 GHz evaluated in the fat in the case (6, 5, 5, a), i.e., the case in which the maximum average error was calculated (Table 3). Colour scale in dB_max_ (0; −50 dB).

Figure 11 shows the root mean squared (RMS) electric field calculated at 0.5 GHz when the probe is immersed in the fat with a height of 70 mm, a width of 15 mm all around the probe, and the probe is inserted up to a 20 mm depth (i.e., the reference condition). For comparison, Figure 12 shows the RMS electric field in the case of a sample height of 6 mm, width 5 mm, and insertion depth 5 mm (i.e., 6; 5; 5; air). From the figures it is evident that, when the tissue sample has greater dimension, the electric field emitted by the open end of the probe is wholly confined within the MUT. On the contrary, if the sample’s dimensions are reduced with respect to the probe’s dimension, frequency, and complex permittivity of the material, then the electric field goes beyond the MUT. This condition is not taken into account in the equivalent circuit used in the reconstruction algorithm, so that the error in the evaluation of the complex permittivity of the material is increased.

## 4. Discussion

In this paper, the experimental set-up used to measure dielectric properties of biological tissues based on the open-ended probe technique was numerically studied to evidence the influence of the dimension of the MUT and location of the probe on the measured data.

The study was motivated by the increasing number of experimental studies devoted to the dielectric characterization of biological tissues, which look for the influence on the dielectric properties of factors as the temperature, blood perfusion, ex vivo vs. in vivo, healthy vs. diseased tissue, and so on [21,45,48]. Such attention is in turn driven by the ever-increasing proposal of medical applications of electromagnetic fields, both as diagnostic tools and therapeutic agents [1,2,3,4,5,6]. Given the requirement for the measurement of slight changes in the dielectric properties and the availability of human samples which are very often limited in dimensions, the measurement error control is mandatory to prevent biasing the dielectric characterization of subtle tissue changes with variability in the experimental set-up.

The study was conducted representing into the CST software the slim-form open-ended coaxial probe and evaluating through the electromagnetic simulation and application of the S&S reconstruction algorithm, the dielectric properties of liver and fat in the frequency range from 0 to 5 GHz. The sample under test was represented by a parallelepiped with varying dimensions; the insertion depth of the probe was also varied. The reconstructed complex permittivity was then compared with the dielectric properties of MUT as implemented in the software, thus deriving a true error related to the performed procedure.

With the aim of validating the numerical model, the first case considered studies a MUT with very big dimensions as well as a very deep insertion depth of the probe (L = 70 mm; w = 15 mm; h = 20 mm) with respect the minimum values reported by the experimental guidelines [34]. Results show an average percentage error, evaluated averaging among the frequency band 100 MHz–5 GHz, of 1.9% (st. dev. 2.0) for the real part and 2.1% (st. dev. 1.7) for the imaginary part of the complex permittivity of liver, and 1.3% (st. dev. 1.0) and 4.6% (st. dev. 2.9), respectively in the case of fat (Table 2; Table 3, case 1). Comparing these values with those reported in the Keysight’s manual, where it is stated that the open-probe should be able to achieve a 5% accuracy in the reconstruction of the dielectric properties of tissue, it can be stated that the numerical evaluation of the complex permittivity of liver and fat conforms to the experimental practice.

Changing the dimensions of the MUT, i.e., reducing the sample’s dimensions, and reducing the insertion depth of the probe with respect the reference condition, the error increases in most of the considered cases (Table 2 and Table 3). As a general comment, the error in the reconstruction of the imaginary part of the complex permittivity is always greater than the error in the real part. Since the error presents as an underestimation of the data, as evidenced in Figure 7 and Figure 9, this means that the model underestimates the dielectric losses of the material. Looking at the different cases, it was noted that the minimum requirement for the experimental studies, i.e., the availability of a 5 mm tissue all around the probe (that means 5 mm insertion depth, and 5 mm distance between the probe and the surfaces of the MUT in all directions) increase the error to 2.8% for $\varepsilon^{\prime }$ and 4.8% for $\varepsilon^{\prime \prime }$ in the case of liver, and to 1.6%, and 8.1%, respectively, in the case of fat (Table 2 and Table 3 case 6). Accordingly, for a setup with minimum dimensions and probe insertion, a 5% accuracy is achievable for a MUT with high water-content, while the imaginary part of the complex permittivity of low water-content tissues shows a much higher error. However, it should be noted that this value represents an average estimate among the frequency band considered; accordingly, the errors will be greater than the 5% value at some frequencies, which, in the case of low-water content tissue, mainly correspond to the higher part of the considered spectrum.

Results displayed on Table 2; Table 3 show that the set-up geometry is critical in different ways for materials with high and low water-content. In particular, it can be noted that the insertion depth of the probe is the most critical parameter which affects the accuracy of the reconstructed complex permittivity of a MUT with high water content. Comparing results from a sample of 70 mm height, 15 mm width in which the probe is placed in contact with the surface (0 insertion depth) with those of a sample 6 mm high, 5 mm wide, in which the probe is inserted for 1 mm only (Table 2, case 4 vs. 17), it can be noted that the error is greater in the bigger sample for both the real and imaginary part of the complex permittivity (2.8 vs. 2.5; 9.4 vs. 6.1). Besides, the arrangements with the probe placed on the surface of the tissue (Table 2, cases 4 and 5) show an average error above the 5% threshold. To derive a measurement guideline, from the data in Table 2 it can be stated that to keep the 5% accuracy in the case of high water content tissues, as liver, the probe should be inserted for at least 3 mm into the sample and at least 3 mm of tissue should be present between the tip of the probe and the bottom surface of the sample. With reference to the width, at least 4 mm are needed all around the probe. The need for a minimum insertion depth and width of the tissue can be explained by the difference in dielectric properties between the high water-content tissue and the surrounding air which cause reflections and distortions of the electric field at the boundary of the tissue. In this respect, it can be noted here that the modelled probe is not equipped with a flange. Although the flange would help in the confinement of the fringe field within the sample, it is not a suitable geometry for minimally invasive measurements of biological tissues. The low distance between the probe tip and the bottom surface of the probe is explained by the high imaginary part of the complex permittivity, i.e., high dielectric losses, which rapidly reduce the electromagnetic field within the sample, thus preventing reflections from the bottom surface. As the probe outer diameter is equal to 2.2 mm, it is found that the width of the sample should be at least 4 times the probe radius to be able to confine the fringe field of the probe and thus allow reliable measurements. Therefore, in case of high water-content tissues, it is advisable to keep the minimum requirements as in the measurement guidelines of at least 5 mm all around the probe.

Looking at the average errors calculated in the case of fat (Table 3), it is immediately noted that the average error in the imaginary part of the complex permittivity is higher than 5% in most of the cases. It is well known that low water-content materials show a greater measurement error [49]. From the values in Table 2, it is derived that the 5% accuracy can be reached only if the greatest dimensions are used for the sample (Table 2, cases 1 and 4). In this respect, it is interesting to note that the minimum error on ε″ is achieved even if the probe is simply in contact with the upper surface of the sample, with no insertion (Table 2, case 4). This result can be explained with the low values of the complex permittivity of fat (Figure 5), that imply a low discontinuity at the boundary between the tissue and air in correspondence of the fringe field of the probe. However, as soon as the width of the MUT or the distance between the probe tip and the bottom surface of the MUT are reduced, the average error increases well above the 5% threshold. In this respect, it is to be noted that the minimum dimension of the MUT required in measurements guidelines, i.e., 5 mm of tissue all around the probe, gives an average error in the considered frequency band up to 8.1% for the imaginary part of the complex permittivity. Similarly, simply reducing the sample width to 5 mm (Table 2, case 3), or lowering the sample height so that the distance between the probe tip and the bottom surface of the sample is 5 mm or lower (Table 2, case 2) increases the average error over the 5% threshold. This result can be explained with the low losses associated with low water-content tissues, which induce a low attenuation of the electric field within the tissue. This in turn allows greater incident electromagnetic energy on the bottom surface of the sample, giving rise to reflections and distortions of the electric field, as evidenced in Figure 12. Therefore, in case of low water-content tissues, the insertion depth of the probe is not critical, and the probe could even be simply placed in contact to the surface of the MUT; however, 5 mm of tissue in all directions with respect to the probe position are a too small dimension to allow accurate measurements. The simulations performed show that a 15 mm dimension would assure the average error to be below 5%. However, noting that both the wavelength and the penetration depth in fat are about 3 times those in liver (e.g., at 5 GHz the wavelength is 3.8 cm in fat vs. 1.3 cm in liver; the penetration depth is 6.7 mm in fat and 2.2 mm in liver) it can be speculated that a 10 mm distance between the probe and the boundary of the sample could be sufficient to achieve the desired accuracy. As a matter of fact, to confirm this conclusion, simulations were performed varying the dimension of the MUT in order to place one boundary of the tissue at a maximum distance of 10 mm from the probe. The different cases as well as the calculated average errors for the real and imaginary part of the complex permittivity are reported in Table 4. From the data in Table 4, it can be seen that the average error is below the chosen threshold of 5% in all cases but the one with the minimum dimensions and in which the probe is touching the upper surface of the MUT.

In [30] the changes in the reflection coefficient of an open-ended probe with flange were studied by way of FDTD method for lossless material and water. The authors concluded that, for biological materials, a sample’s thickness of about twice the aperture of the probe is needed. From the results in this study, it is seen how this conclusion is valid for high losses materials, as the liver, which show dielectric properties close those studied in [30]; however, the same doesn’t apply for low loss materials as fat. Finally, in both liver and fat, if the MUT is placed on a conductive support, there is a slight reduction of the average error compared with the cases with the same MUT dimensions backed with air (6 vs. 19, and 15 vs. 20). To this end, it should be noted that the MUT dimensions in the PEC cases are the minimum experimental requirements (case 19) and the minimum dimensions to still maintain the 5% accuracy in the case of liver (case 20). In any case, if the material used to hold the sample influences the achieved results, then the measurement is not probing only the MUT.

The limitations of this study are linked to the use of the numerical solution to derive the reflection coefficient of the probe, so that the calculated errors include the numerical accuracy, also. Indeed, in the experimental case, the accuracy of the measure is influenced by the MUT, as investigated in this study, and by the uncertainty of the measure. An additional point to be noted is related to the contact between the probe and the MUT. In the numerical study the tissue conforms to the probe, without air gaps, both when the probe is inserted in the MUT and when it is placed on its surface. This condition is more critical in the experimental case, where the presence of air bubbles or the accumulation of fluids in the interstitial space cannot be completely ruled out. Moreover, the procedure used to try to guarantee the proper contact between the probe and the tissue by pressing the MUT against the tip of the probe can even worsen the situation, by piping biological fluids, such as serum and blood, towards the location of the probe tip. Finally, the achieved results are strictly related to the method used to reconstruct the unknown properties from the measured reflection coefficient. More complex methods, as those based on modal expansion, could achieve better performances than those here reported [29]. It is worth noting here that the reported results substantially confirm the good practice requirements of common experimental set-up [34].

Once the errors induced on the measured dielectric properties by small MUT have been characterized, other factors influencing the result of the measure could be considered, as different temperature values, the presence of small air gaps between the tip of the probe and the materials, as well as the not homogeneity of the sample.

As a concluding comment, it can be noted that once the numerical analysis is validated, as in this study, it can be a valid tool to assess the suitability of a measurement set-up for a target accuracy threshold.

## 5. Conclusions

In the measurements of dielectric properties of tissues by way of the open-ended probe technique, the location of the tip of the probe and the dimension of the sample under test are essential for achieving high accuracies. However, sample’s dimensions and probe’s tip locations often are dictated by the experimental condition, especially in in vivo cases. The performed study showed the applicability of a numerical analysis with a reference dielectric truth to calculate, prior to or after the measurement, the error due to the reconstruction method for the specific set-up conditions (sample size and probe positioning). Additionally, the numerical sensitivity investigation carried out on liver and fat tissues showed the most critical factors in the set-up geometry for these MUTs, differentiating the errors according the expected properties of the MUT itself (i.e., high and low water content tissues). In particular, the study showed that when high water-content tissues are under measure, the tip of the probe should be inserted into the sample for at least 5 mm, and the sample dimensions should assure at least 5 mm all around the probe. When low water-content tissues are measured, the probe could be simply placed in contact with the surface of the sample; however, at least 10 mm all around the probe are needed to achieve a 5% accuracy in the measure.

## Figures and Tables

**Table 1:** Cases considered in the sensitivity analysis.

N	Case	Height (L mm)	Width (w, mm)	Insertion Depth (h, mm)	Backing
1	reference	70	15	20	air
2	shorter	25	15	20	air
3	narrow	70	5	20	air
4	0 insertion	70	15	0	air
5	shorter + narrow + 0 ns.	25	5	0	air
6	influence of the height (narrow, small insertion depth)	10	5	5	air
7		9	5	5	air
8		8	5	5	air
9		7	5	5	air
10		6	5	5	air
11	influence of the width (short, small insertion depth)	10	4	5	air
12		10	3	5	air
13		10	2	5	air
14	influence of the insertion depth (short, narrow)	9	5	4	air
15		8	5	3	air
16		7	5	2	air
17		6	5	1	air
18		5	5	0	air
19	influence of the support	10	5	5	PEC ^1^
20		8	5	3	PEC ^1^

^1^ PEC = Perfect Electric Conductor.

**Table 2:** Average errors achieved in the evaluation of the real and imaginary part of the relative complex permittivity of liver in several geometrical conditions.

N	Case Name (L; w; h; Backing)	$\varepsilon^{\prime }\;\mathrm{Error}$ Average (st. dev)	$\varepsilon^{\prime \prime }\;\mathrm{Error}$ Average (st. dev)
1	reference (70; 15; 20; air)	1.9 (2.0)	2.1 (1.7)
2	shorter (25; 15; 20; air)	2.1 (2.1)	2.4 (1.8)
3	narrow (70; 5; 20; air)	2.6 (2.5)	3.3 (2.5)
4	0 insertion (70; 15; 0; air)	2.8 (2.2)	9.4 (3.9)
5	s + n + 0 ins (25; 5; 0; air)	2.9 (2.7)	10.3 (3.7)
6	height (10; 5; 5; air)	2.8 (2.8)	4.8 (2.9)
7	height (9; 5; 5; air)	2.8 (2.8)	4.6 (2.7)
8	height (8; 5; 5; air)	2.5 (2.8)	4.3 (2.7)
9	height (7; 5; 5; air)	1.7 (3.0)	6.8 (3.1)
10	height (6; 5; 5; air)	11.1 (3.0)	19.6 (4.3)
11	width (10; 4; 5; air)	2.9 (2.9)	4.9 (2.6)
12	width (10; 3; 5; air)	1.8 (3.0)	9.0 (3.7)
13	width (10; 2; 5; air)	8.8 (3.1)	17.6 (4.2)
14	insertion (9; 5; 4; air)	3.1 (2.9)	5.2 (3.9)
15	insertion (8; 5; 3; air)	3.4 (2.9)	5.0 (3.2)
16	insertion (7; 5; 2; air)	3.2 (3.0)	4.7 (2.3)
17	insertion (6; 5; 1; air)	2.5 (3.0)	6.1 (2.6)
18	insertion (5; 5; 0; air)	3.0 (3.3)	10.7 (3.4)
19	support (10; 5; 5; PEC)	1.9 (2.6)	4.6 (2.8)
20	support (8; 5; 3; PEC)	2.1 (2.9)	4.9 (3.3)

**Table 3:** Average errors achieved in the evaluation of the real and imaginary part of the relative complex permittivity of fat in several geometrical conditions.

N	Case Name (L; w; h; Backing)	$\varepsilon^{\prime }\;\mathrm{Error}$ Average (st. dev)	$\varepsilon^{\prime \prime }\;\mathrm{Error}$ Average (st. dev)
1	reference (70; 15; 20; air)	1.3 (1.0)	4.6 (2.9)
2	shorter (25; 15; 20; air)	1.5 (1.0)	5.2 (3.5)
3	narrow (70; 5; 20; air)	1.8 (1.0)	6.5 (3.8)
4	0 insertion (70; 15; 0; air)	2.8 (1.0)	4.6 (3.5)
5	s + n + 0 ins (25; 5; 0; air)	3.0 (1.0)	5.6 (3.6)
6	height (10; 5; 5; air)	1.6 (0.9)	8.1 (4.5)
7	height (9; 5; 5; air)	0.9 (0.7)	8.5 (4.6)
8	height (8; 5; 5; air)	2.0 (0.9)	9.2 (4.7)
9	height (7; 5; 5; air)	3.4 (0.9)	11.4 (4.8)
10	height (6; 5; 5; air)	10.7 (0.8)	21.0 (4.7)
11	width (10; 4; 5; air)	2.0 (0.9)	8.6 (4.5)
12	width (10; 3; 5; air)	2.8 (0.9)	9.4 (4.4)
13	width (10; 2; 5; air)	6.6 (1.1)	12.9 (3.4)
14	insertion (9; 5; 4; air)	1.6 (0.9)	8.1 (4.5)
15	insertion (8; 5; 3; air)	1.7 (0.9)	8.1 (4.6)
16	insertion (7; 5; 2; air)	1.9 (0.9)	8.0 (4.5)
17	insertion (6; 5; 1; air)	2.1 (0.8)	7.7 (3.7)
18	insertion (5; 5; 0; air)	3.1 (1.0)	6.9 (3.4)
19	support (10; 5; 5; PEC)	1.2 (0.7)	6.2 (3.4)
20	support (8; 5; 3; PEC)	1.3 (0.7)	6.2 (3.8)

**Table 4:** Average errors achieved in the evaluation of the real and imaginary part of the relative complex permittivity of fat when the MUT has one boundary at 10 mm from the probe.

N	Case L; w; h; Backing	$\varepsilon^{\prime }\;\mathrm{Error}$ Average (st. dev)	$\varepsilon^{\prime \prime }\;\mathrm{Error}$ Average (st. dev)
1	70; 10; 20; air	0.8 (0.8)	4.5 (3.3)
2	30; 15; 20; air	0.9 (0.8)	4.7 (2.9)
3	30; 10; 20; air	0.9 (0.8)	4.6 (3.3)
4	10; 10; 0; air	2.8 (1.2)	6.1 (4.7)
